# Image-based food monitoring and dietary management for patients living with diabetes: a scoping review of calorie counting applications

**DOI:** 10.3389/fnut.2025.1501946

**Published:** 2025-03-27

**Authors:** Asal Rouhafzay, Ghazal Rouhafzay, Jalila Jbilou

**Affiliations:** ^1^School of Psychology, Université de Moncton, Moncton, NB, Canada; ^2^Centre de formation médicale du Nouveau-Brunswick, Université de Moncton, Moncton, NB, Canada; ^3^Department of Computer Science, Université de Moncton, Moncton, NB, Canada

**Keywords:** calorie estimation, image segmentation, volume estimation, diabetes management, methodology, image classification, scoping review, calorie counting apps

## Abstract

Accurate dietary intake estimation is crucial for managing weight-related chronic diseases, such as diabetes, where precise measurement of food volume and caloric content is essential. Traditional calorie counting methods are often error-prone and may not meet the specific needs of individuals with diabetes. Recent advancements in computer science offer promising solutions through automated systems that estimate calorie intake from food images using deep learning techniques. These systems provide personalized dietary recommendations, helping individuals with diabetes make informed choices. As smartphones and wearable devices become more accessible, the utilization of electronic apps for dietary monitoring is increasing, highlighting the need for more research into safe, secure, and evidence-based IoT solutions. However, challenges such as standardization, validation across diverse populations, and data privacy concerns need to be addressed. This review focuses on the role of computer science in dietary intake estimation, specifically food segmentation, classification, and volume estimation for calorie calculation. By synthesizing existing literature, this review provides insights into current methods, key challenges, and potential future directions. The review also explores advancements in technology that can improve the accuracy of dietary assessments, contributing to personalized disease management and the prevention of weight-related chronic conditions.

## 1 Introduction

Weight-related diseases, including diabetes, are labeled as a pandemic and represent an alarmingly increasing global public health issue. The prevalence of diabetes has tripled these last 15 years, rising more rapidly in low-and middle-income countries than in high-income countries. In 2021, approximately 537 million adults worldwide were living with diabetes, a figure expected to rise to 783 million by 2045 if current trends continue (1). Diabetes is a major cause of serious health complications, including blindness, kidney failure, heart attacks, strokes, and lower limb amputations. There are different types of diabetes, with Type 2 Diabetes (T2D) being the most prevalent and largely preventable. Managing T2D involves adopting healthy behaviors such as following a balanced diet (particularly low in carbohydrates and fat), engaging in regular physical activity, and, when necessary, taking medication. Consistent medical follow-ups are also essential for effective T2D management.

Managing diabetes effectively requires accurate monitoring of dietary intake, particularly caloric consumption (2). Traditional methods, such as food diaries and self-reporting, have long been used to estimate dietary intake (3). However, these methods are liable to errors due to underreporting, overreporting, and recall biases, which can significantly impact the accuracy of calorie calculations (4). This is especially problematic for individuals with diabetes, where precise management of caloric intake is crucial to maintaining stable blood glucose levels.

Recent advancements in computer science, particularly in the fields of artificial intelligence (AI) and computer vision, have introduced innovative solutions to these challenges. By leveraging deep learning algorithms, researchers have developed systems that can automatically segment, classify, estimate food volume and caloric content from images, eliminating the need for manual entry and reducing the potential for human error. These approaches offer the potential to revolutionize dietary monitoring by providing accurate, real-time assessments of food intake (5).

Previous research in this domain has explored various methods for improving dietary intake estimation, including the use of specialized hardware, such as 3D scanners and depth sensors, to capture more accurate food measurements (6). While these methods have shown promise, their reliance on specialized equipment limits their accessibility and widespread adoption. In contrast, image analysis can now be performed using standard smartphone cameras, thanks to everyday developments and improvements in smartphone technology, making these solutions more accessible and practical for daily use.

Theories surrounding personalized medicine and precision health underscore the importance of tailoring interventions to individual needs (7). In the context of diabetes management, this means providing dietary recommendations that align with a person’s specific metabolic profile, dietary habits, lifestyle, knowledge and capacity. AI-driven dietary monitoring tools align well with these theories by enabling more personalized and adaptive approaches to diabetes care.

However, despite the promise of these technologies, several challenges remain, specifically in achieving accurate automated volume estimation without user input or specialized devices. Additionally, issues related to the standardization of methods, validation across diverse populations, and privacy concerns in handling sensitive health data must be addressed to ensure the reliability and ethical use of dietary monitoring tools (8).

Given the critical role of accurate dietary monitoring in diabetes management and the rapid advancements in AI and computer vision, this paper provides a comprehensive review of 14 popular in the market calorie-counting applications. It critically evaluates the computer science methodologies employed in their development, focusing on food segmentation, classification, volume estimation, and calorie calculation. By synthesizing recent advancements introduced through reputable platforms such as IEEE, Springer, and ACM, this review emphasizes the technological innovations driving more accurate and personalized dietary assessments. It serves as a foundation for developing next-generation calorie-counting tools, offering insights into the strengths and limitations of current approaches and paving the way for future research and application development. The aim of the current paper is to identify solutions offered in the form of mobile applications, whose working principles are publicly available and can be studied. The aim of this paper is to critically appraise the existing literature on calorie-counting applications. It seeks to extract and evaluate the computer science methodologies employed, including food segmentation, classification, volume, and calorie estimation. Additionally, it compares the effectiveness and accuracy of these methodologies across various applications and derives recommendations for practical use and future research.

## 2 Materials and methods

This review synthesizes literature from several well-established computer science databases, including IEEE, Springer, ACM, and ScienceDirect, to evaluate advancements in calorie-counting applications. The focus was on image-based food monitoring systems and calorie-counting tools, both manual and automated, that utilize computer science methodologies such as food segmentation, classification, and volume estimation to enhance dietary intake accuracy. These tools are particularly relevant for individuals managing weight-related chronic diseases like diabetes.

### 2.1 Search terms and databases

Key search terms were carefully crafted based on initial scoping exercises and included combinations of keywords: “calorie counting apps,” “food image segmentation,” “food volume estimation,” “Image Processing” “dietary intake estimation,” and “diabetes management.” Search was limited to studies and applications published or introduced between 2010 and 2024 to focus on advancements spanning the past 15 years.

### 2.2 Article retrieval and screening protocols

Articles and application descriptions were retrieved through queries across databases. A multi-step screening process was implemented, beginning with the review of titles and abstracts to identify relevant studies. Full-text analysis was conducted to ensure methodological transparency and relevance to calorie-counting tools. Duplicates and irrelevant studies were excluded during this process.

### 2.3 Inclusion and exclusion criteria

Studies and applications were included if they explicitly focused on calorie-counting tools, whether manual or automated, and presented the computer science methodologies employed in their design, such as food segmentation, classification, or volume estimation. Excluded were those lacking sufficient methodological detail, not within the specified time range, or irrelevant to dietary monitoring.

### 2.4 Selection of most frequently studied applications

**Figure 1:**
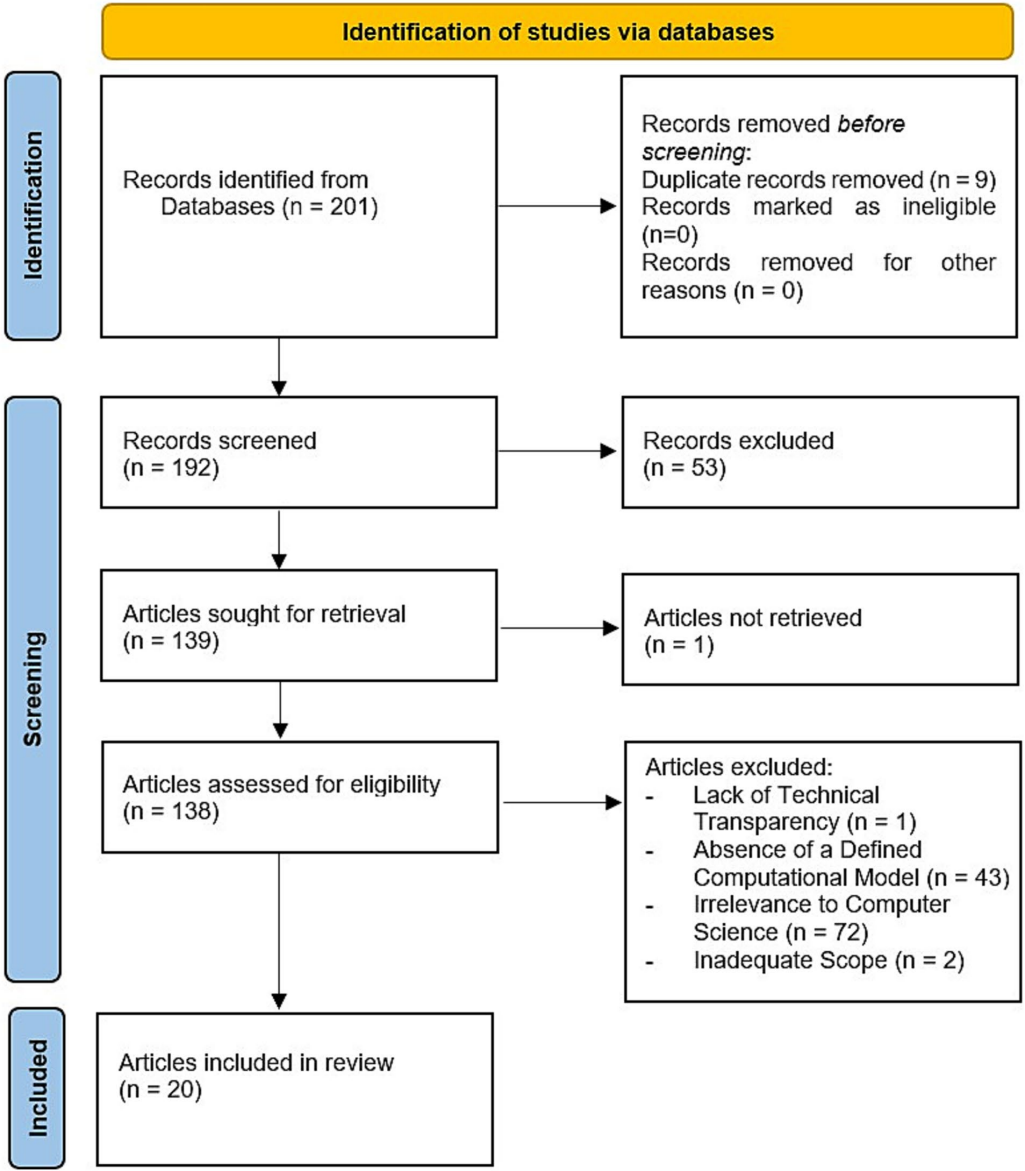
PRISMA flow diagram.

The 14 calorie-counting applications analyzed in this review represent a mix of manual, semi-automated, and AI-driven tools. To select the most studied calorie-counting applications, we used the following criteria: 1-frequent citation in the literature (more than 3 different articles), 2-availability of public documentation describing the methodologies used, and 3-contributions to advancing dietary monitoring practices. By including both manual and automated tools, the review provides a comprehensive overview of the progression and diversity in calorie-counting methodologies. Figure 1 provides a flow diagram summarizing the screening and selection process for identifying the 14 applications included in this review.

This approach allowed for an in-depth analysis of advancements in calorie-counting applications, along with an evaluation of their limitations, reliance on user input, sensitivity to image quality variations, and scalability challenges. These findings aim to highlight areas where future research can further improve the accuracy and accessibility of these tools.

## 3 Core stages and working principles of calorie counting apps

This section provides a detailed analysis of the 14 prominent calorie-counting applications selected for this review, as introduced in the previous section. These applications, highlighted for their contributions to computer science methodologies and were released through prominent publishers. We explore the main stages involved in calorie counting applications, focusing on three critical steps: food segmentation, food recognition, and food volume estimation. These steps are fundamental to the accurate calculation of nutritional information and calorie content from food images. Additionally, these applications often rely on well-known food datasets, which play a crucial role in ensuring the accuracy and comprehensiveness of food recognition and calorie estimation.

**Figure 2:**
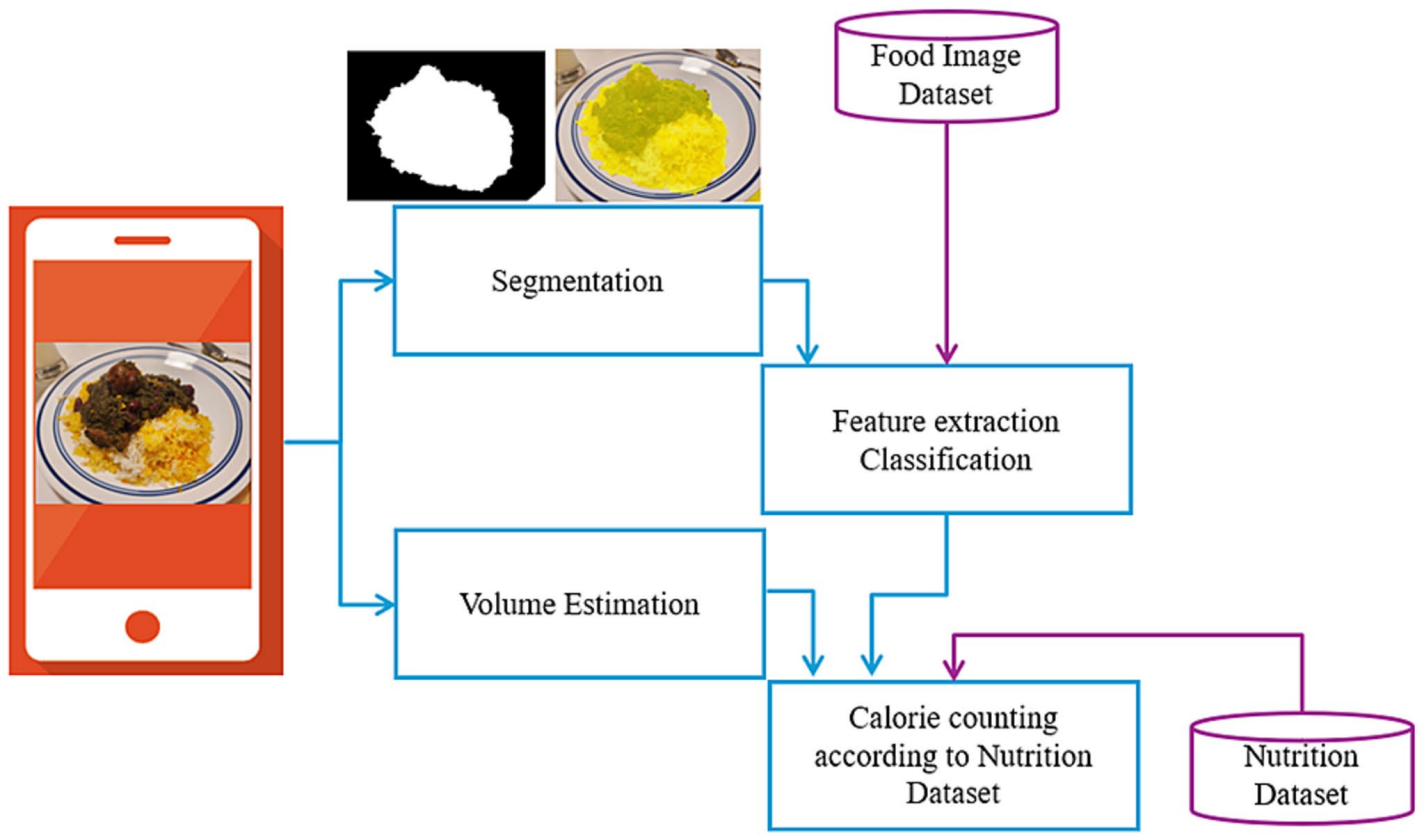
An automated image-based nutrition assessment tool.

By analyzing these stages across the 14 applications, we aim to identify trends, strengths, and potential areas for improvement in the current state of calorie-counting technology. This analysis provides insights into how each application approaches the challenges of food segmentation, recognition, and volume estimation, all of which are crucial for accurate calorie calculation and effective dietary monitoring. The block diagram in Figure 2 illustrates the core stages involved in the calorie estimation process. The process begins with food image segmentation, where food items are isolated from the background or other objects in the image. Following segmentation, food classification identifies the specific type of food, such as distinguishing between white rice, brown rice, or meat. The classified food items then undergo volume estimation, where their portion sizes are calculated using appropriate techniques. Finally, the results of classification and volume estimation are integrated with nutritional datasets to perform calorie counting, determining the caloric value of the food items. These tasks are sequentially dependent, with each step building upon the previous one to achieve accurate dietary assessment. Table 1 presents the selected applications and provides their general information.

**Table 1:** The selected 14 calorie counting applications.

Year	App	Author	Country	Affiliation	Application Focus
2011	PlateMate (39)	Jon Noronha et al.	USA	Harvard	Calorie estimation through annotations from nonexpert Amazon Mechanical Turk workers
2013	FoodLog (37)	Aizawa et al.	Japan	University of Tokyo	Personal dietary management via mobile app
2015	Snap-n-Eat (36)	Zhang et al.	USA	SRI International, Princeton	Real-time food recognition
2015	Menu-Match (42)	Beijbom et al.	USA	University of California	Matching food images to menu items
2015	Im2Calories (7)	Meyers et al.	USA	Google	Automated calorie estimation
2015	FoodCam (10, 43)	Kawano et al.	Japan	UEC	The system is designed to operate entirely on a smartphone without needing to send data to external servers, leveraging the computational power of modern mobile devices for real-time recognition.
2016	GoCarb (44, 45)	Rhyner et al.	Switzerland	U of Bern	Carb counting for diabetic patients
2017	NU-InNet (46)	Termritthikun et al.	Thailand	Naresuan University	Thai-food recognition application in a smartphone
2018	DietLens (38)	Ming et al.	Singapore	University of Singapore	Comprehensive dietary assessment
2019	Food Tracker (47)	Jianing Sun et al.	Canada	McGill University	Mobile food tracking
2020	MyDietCam (48)	Tahir and Loo	Malaysia	University of Malaya	Real-time dietary monitoring
2020	goFOOD™ (29)	Lu et al.	Switzerland	University of Bern	Food portion estimation
2020	DeepFood (49)	Jiang et al.	Canada	MacGill University	Food recognition and dietary assessment system
2021	Mobile food record (mFR)	Shao et al. (50)	USA	Purdue University	Dietary assessment in research settings

### 3.1 Food image datasets

Food image datasets are foundational for the development and evaluation of food recognition systems. These datasets vary widely in their attributes, including the number of images, food categories, and methods of data acquisition. A well-structured food image database is critical for training and benchmarking machine learning models, impacting their performance and generalizability.

Food image datasets are categorized by several factors. Different datasets focus on various food types, ranging from generic classifications to specific cuisines. For example, datasets such as *Food-101* (9) and *UEC-Food256* (10) cover a broad spectrum of food categories, while others, like *Turkish-Foods-15* (11) and *Japanese Foods* (10, 12–16), focus on specific regional cuisines. Also, the source and method of image collection play a significant role in the quality and applicability of the database. Images may be captured in controlled environments, such as studios with standardized lighting, or in natural settings, like restaurants and social media platforms. For instance, *Food-85* (17) and *Diabetes* (18) use controlled environments, whereas *Foodlog* (19) and *Instagram 800k* (20) leverage user-contributed images and web crawls.

The number of images and their diversity within each class are crucial for model robustness. Datasets like *FoodX-251* (21) and *Fruits 360 Dataset* (22) offer extensive image collections, which are essential for training deep learning models. High diversity in images helps the model generalize better to new, unseen data. Food image datasets are often designed for specific tasks, such as classification or segmentation. For example, *FOOD201-Segmented* (7) contains images specifically segmented for classification tasks, while datasets like *VIREO Food-172* (23) may serve both classification and segmentation needs. NutriNet (24) is another influential database designed for deep learning applications in food and drink image recognition. It plays a pivotal role in dietary assessment and nutritional analysis, further enhancing AI’s capabilities in health informatics.

Recent food image datasets like CNFOOD-241 (25), AI4FoodDB (26), and MyFoodRepo-273 (27) have made significant contributions to the field. AI4FoodDB (26), launched in 2023, is particularly notable as it forms part of a larger initiative aimed at advancing personalized nutrition and e-Health solutions. What sets AI4FoodDB apart is its integration of food images with data from wearable devices, validated questionnaires, and biological samples. This holistic approach seeks to create a digital twin of the human body, providing a valuable benchmark for personalized nutrition research and aiding in the fight against non-communicable diseases.

As mentioned, many existing food image datasets are predominantly focused on specific countries or cultural contexts, which can introduce significant biases in the development of food recognition models and constrain their generalizability across diverse dietary habits globally. The limited cultural diversity in these datasets often results in AI systems that underperform when encountering food items from underrepresented regions or cuisines. This lack of inclusivity poses a critical challenge to the development of robust, globally applicable dietary assessment tools. To address this, there is a pressing need for the creation of comprehensive datasets that capture the breadth of global food practices. Initiatives such as AI4FoodDB, which integrate diverse food categories alongside multimodal data sources, exemplify a forward-looking approach to enhancing model generalization and reducing biases in food recognition systems.

Table 2 provides a summary of notable food image datasets, highlighting their unique attributes.

**Table 2:** Food image datasets.

Authors	Year	Dataset	Food Category	Images/Class	Image Source
Godwin et al. (51)	2006	Wedge Shape foods dataset	American Foods	3 categories	Controlled environment
Chen et al. (52)	2009	PFID	American Fast Foods	1,038 (16)	Fast food data captured in multiple restaurants
Mariappan et al. (53)	2009	TADA	Artificial And Generic Food	256 (11)	Controlled environment
Yanai et al. (17)	2010	Food-50	Japanese Foods	5,000 (50)	Crawled from web
Hoashi et al. (17)	2010	Food-85	Japanese Foods	8,500 (85)	Existing food datasets
Miyazaki et al. (19)	2011	Foodlog	Japanese Foods	6,512 (2,000)	Captured by users
Marc Bosch et al. (54)	2011	FNDDS	American Foods	7,000	Images of food accquired by users
Chen et al. (55)	2012	ChineseFoodNet	ChineseFood	192,000 (208)	Gathered from web
Matsuda et al. (16)	2012	UECFOOD-100	Japanese Foods	14,361 (100)	Captured by mobile camera
Chen et al. (55)	2012	Chen	Chinese Foods	5,000/50	Crawled from the Internet
Anthimopoulos et al. (18)	2014	Diabetes	European	5,420 (11)	Controlled environment and downloaded from the web
Bossard et al. (9)	2014	Food-101	American Foods	101,000 (101)	Crawled from web
Bossard et al. (9)	2014	ETHZ Food-101	American Foods	100,000 (101)	Crawled from web
Kawano et al. (10)	2014	UECFOOD-256	Japanese Foods	25,088 (256)	Captured by mobile camera
Stutz et al. (56)	2014	Rice dataset	Generic (Rice)	1 food type	Acquired from user
Farinella et al. (57)	2014	UNCIT-FD889	Italian Foods	3,583 (899)	Acquired with a smartphone
Myers et al. (7)	2015	FOOD201-Segmented	American Foods	12,625	Manually annotated dataset
Myers et al. (7)	2015	Restaurant	-	2,517	web images for 23 restaurants
Myers et al. (7)	2015	Gfood3D	(everyday) foods for 3D volume estimation	-	50 Google meals, captured by Google using a dedicated setup for 3D food modeling
Myers et al. (7)	2015	Nfood3D	-	-	11 meals made with Nasco food replicas
Xin Wang et al. (58)	2015	UPMC Food-101	Generic	100,000 (101)	Crawled from web
Cioccoa et al. (59)	2015	UNIMB 2015	Generic	2,000 (15)	Using a Samsung Galaxy S3 smartphone
Fang et al. (60)	2015	TADA	American Foods	19 categories	Controlled environment
Herranz et al. (61)	2015	Dishes	Chinese Restaurant Foods	117,504 (3,832)	Download from dianping
Beijbom et al. (42)	2015	Menu-Match	Generic Restaurant Food	646 (41)	Captured from social media
Zhou et al. (62)	2016	Food-975	Chinese Foods	37,785 (975)	Collected from restaurants
Chen et al. (23)	2016	Vireo-Food 172	Chinese Foods	110,241 (172)	Downloaded from web
Cioccoa et al. (5)	2016	UNIMB 2016	Italian Foods	1,027 (73)	Captured from dining tables
Bolaños et al. (63)	2016	EgocentricFood	Generic	5,038 (9)	Taken by a wearable egocentric vision camera
Wu et al. (64)	2016	Food500	Generic	148,408 (508)	Crawled from web
Singla et al. (65)	2016	Food-11	Generic	16,643 (11)	Other food datasets
Farinella et al. (66)	2016	UNCIT-FD1200	Generic	4,754 (1,200)	Acquired using smartphone
Jaclyn Rich et al. (20)	2016	Instagram 800 k	Generic	808,964 (43)	Social media
Liang et al. (67)	2017	ECUSTFD	Generic	2,978 (19)	Acquired using smartphone
Mezgec et al. (24)	2017	NutriNet	Generic	225,953 (520)	Downloaded from web
Güngör et al. (11)	2017	Turkish-Foods-15	Turkish Dishes	7,500 (15)	Collected from other datasets
Pandey et al. (68)	2017	Indian Food Database	Indian Foods	5,000 (50)	Downloaded from web
Termritthikun et al. (69)	2017	THFood-50	Thai Foods	700/50	Downloaded from web
Ciocca et al. (70)	2017	FOOD524DB	Generic	247,636 (524)	Existing food database
Hou et al. (71)	2017	VegFru	Fruit and VEG	160,731 (292)	Collected from search engine
Waltner et al. (72)	2017	FruitVeg-81	Fruit and VEG	15,630 (81)	Collected using mobile phone
Muresan et al. (22)	2018	Fruits 360 Dataset	Fruit Dataset	71,125 (103)	Camera
Qing Yu et al. (15)	2018	FLD-469	Japanese Foods	209,700 (469)	Smart Phone camera
Ming et al. (38)	2018	Singapore Hawker Dataset	Chinese, Western, Indian, Malay food, snacks, fruits, desserts and beverage etc.	249	Apart from ImageNet Dataset and Google image search.
Donadello et al. (73)	2019	FfoCat	Mediterranean	58,962 (156)	Downloaded from web
Kaur et al. (21)	2019	FoodX-251	Generic	158,000 (251)	Collected from web
Gao et al. (14)	2019	SUEC Food	Japanese Foods	31,395 (256)	Acquired from other datasets
Aguilar et al. (48)	2019	MAFood-121	Spanish Foods	21,175	Google search engine
Ege et al. (13)	2019	UECFoodPix	Japanese Foods	10,000 (100)	Acquired from other datasets
Wang et al. (74)	2019	Mixed dishes	Chinese	12,105 (216)	Captured by authors
Ghalib et al. (48)	2020	Pakistani Food Dataset	Pakistani Foods	4,928 (100)	Crawled from web
Aslan et al. (75)	2020	Food50Seg	Japanese	5,000 (50)	Acquired from other datasets
Konstantakopoulos et al. (76)	2021	MedGRFood	Mediterranean	51,840 (160) 5,000 (190)	Controlled environment and downloaded from web
Wu et al. (77)	2021	FoodSeg103 FoodSeg154	Generic	7,118 (730) 9,490 (730)	Acquired from other datasets
Okamoto et al. (12)	2021	UECFoodPix Complete	Japanese Foods	10,000 (102)	Acquired from other datasets
Mohanty et al. (27)	2022	MyFoodRepo-273	Swiss Foods	24,119 (273)	Crawled from web
Romero-Tapiador et al. (26)	2023	AI4FoodDB	Diverse	Not specified	Self-collected data, smartphone images, and wearable device data
Chen et al. (25)	2024	CNFOOD-241	Chinese Food	191,811 (241)	Gathered from web

While Table 2 focuses on publicly documented, food-focused image datasets, certain calorie-counting applications also reference specialized datasets that do not strictly fit these criteria. For example, Im2Calories (7) utilizes NYU Depth V2 (28) for initial depth training; however, we do not include it here because it is a general-purpose indoor scene dataset rather than a food-specific resource. These cases illustrate that some applications leverage additional or proprietary datasets for specialized tasks, particularly volume estimation, that fall outside the scope of publicly available food-image collections.

### 3.2 Image segmentation

Image segmentation is a foundational technique in computer vision, involving the partitioning of an image into distinct regions or segments that correspond to different objects or areas of interest. In food recognition, segmentation is particularly important because it enables the precise identification and isolation of individual food items on a plate. This accuracy is critical for tasks such as portion size estimation, calorie counting, and nutrient analysis, all of which are essential components of dietary assessment systems.

In food recognition applications, segmentation plays a vital role in ensuring that each food item is accurately identified and analyzed, regardless of how it is presented on the plate. Given the variability in food presentation due to different cuisines, cooking methods, and serving styles, segmentation methods must be robust and adaptable. These methods range from traditional approaches like edge detection and region-based segmentation to advanced deep learning models that can learn complex features from large datasets.

Numerous mobile applications and systems have been developed that incorporate image segmentation as a key component for dietary assessment. These applications often utilize various segmentation techniques, each chosen based on the specific requirements and constraints of the application, such as processing power, real-time capabilities, and the complexity of food items being analyzed. The following Table 3 summarizes the segmentation methods utilized in 14 prominent food recognition applications, detailing their approaches:

**Table 3:** Segmentation strategies employed in food image processing applications.

App	Segmentation Method	Approach
PlateMate (39)	Drawing Boxes: Workers (referred to as Turkers) draw bounding boxes around each distinct food item in a photograph.	This segmentation process is done manually. They used similarity comparison and voting mechanisms to ensure accuracy
FoodLog (37)	Block-Wise Image Analysis—Block Classification	The image is divided into blocks of 16x16 pixels. These blocks are then analyzed using color and frequency features. SVM is used to classify food/ nonfood. Each block is classified into one of six labels: five food categories (grains, vegetables, meat/fish/beans, fruit, dairy products) or “nonfood.” This classification is performed using an AdaBoost classifier
Snap-n-Eat (36)	Saliency-Based Sampling Region-Based Sampling	The system uses a saliency detection algorithm to identify the most visually prominent regions in the image, which are likely to contain food items. This saliency map helps in focusing on the food rather than the background. Hierarchical segmentation is then performed to divide the image into multiple regions. This process involves grouping smaller regions into larger, meaningful segments based on features like color, texture, and size similarity. This hierarchical grouping allows for better discrimination and segmentation of individual food items.
Menu-Match (42)	The paper does not explicitly detail a separate segmentation process. Instead, it focuses on a holistic assessment approach where food items are recognized based on a predefined restaurant-specific database.	This method minimizes the need for traditional segmentation by relying on the consistency of the food items across servings.
Im2Calories (7)	The segmentation method used in the paper involves a CNN-based semantic image segmentation	The system leverages the “DeepLab” model, which uses a CNN to provide the unary potentials of a Conditional Random Field (CRF) and employs a fully connected graph to perform edge-sensitive label smoothing, like bilateral filtering. The model is first pre-trained on ImageNet and then fine-tuned on a custom food dataset (Food201-segmented). The method also includes a global image context, which is provided by a multi-label classifier to improve segmentation performance by reducing false positives.
FoodCam (43)	GrubCut Segmentation: The system first segments the food items using GrubCut to refine the regions within the bounding boxes drawn by the user.	When a user draws bounding boxes around food items on the screen, GrabCut is applied to adjust these boxes, ensuring they accurately fit the food regions. The bounding box adjustment is performed only once after the initial bounding box is drawn to optimize the computational cost, as GrabCut is relatively high in computational demand.
GoCarb (44, 45)	Image Cropping: Color Space Conversion: Filtering: Region Growing: Background and Plate Segmentation: Accuracy: The segmentation method achieved an accuracy of 88.5% when tested on manually annotated food images.	The images are cropped around the detected elliptical plate. The cropped images are converted to the CIELAB color space, which is perceptually uniform. Pyramidal mean-shift filtering is applied to smooth fine-grain textures while preserving dominant color edges. This makes pixels of the same food item have similar colors, distinguishable from others. A region growing algorithm is used to merge pixels of similar colors into segments, producing initial segmentation. Small regions are then merged with their closest neighbors based on color similarity. Segments that belong to the background or plate are identified and discarded based on their location relative to the detected ellipse.
NU-InNet (46)	Segmentation with Convolutional Neural Networks (CNNs)	Although segmentation is not a primary focus, the paper mentions using CNNs to improve food identification by separating the food from the background. This is briefly referenced in relation to enhancing the effectiveness of food recognition, but the paper does not provide a detailed segmentation methodology. Instead, the focus is on image recognition without extensive preprocessing or segmentation to maintain efficiency on mobile devices.
DietLens (38)	Nor specified	DietLens uses a novel photo-based portion selection method where users select from pre-compiled reference images showing different portion sizes of the same food type. These reference photos are linked with nutritional information, such as calorie content. This method, which does not require prior knowledge of portion sizes, acts as a form of segmentation, helping users to visually compare and select the portion size that matches their actual meal.
Food Tracker (47)	YOLOv2 Strategy: The paper uses the YOLOv2 framework for food detection, which involves dividing the input image into a grid and predicting bounding boxes for each grid cell.	K-means clustering box. The bounding boxes contain the predicted class label and coordinates, which localize the food items within the image. Although not explicitly termed as “segmentation,” this process effectively segments the food items from the background by generating bounding boxes around them.
MyDietCam (48)	Not specified	As it appears on the Figure 1 (48) Bonding Box
goFOOD™ (29)	Automatic Segmentation, Mask R-CNN framework used for automatic food segmentation. Semi-Automatic Segmentation	Instance segmentation, which identifies the location and boundaries of food items within an image. Mask R-CNN is used for segmentation due to the cost and impracticality of creating a large, instance-segmented training dataset for many food categories. If the automatic segmentation is unsatisfactory, users can manually indicate each food item on the image. The system then generates a new segmentation map using traditional region growing and merging algorithms, with user input serving as the seed points.
DeepFood (49)	Region Proposal Network (RPN)	The segmentation is performed by generating multiple regions of interest (RoIs) using the Region Proposal Network (RPN) derived from the Faster R-CNN model. These RoIs help to separate the food items from the background, improving the detection model’s efficiency by isolating the relevant food objects.
mobile food record (mFR) (50)	Class-Agnostic Method Super Pixel-Based Segmentation	This method uses a pair of eating scene images to identify salient missing objects without prior information about the food class. Efficient super pixel-based segmentation methods are developed, with some approaches utilizing weak supervision for improved food image segmentation.

This table outlines the variety of segmentation methods employed across different food recognition applications, each tailored to the unique challenges posed by food imagery. For instance, a range of segmentation methods, from manual approaches like PlateMate, where workers manually draw bounding boxes, to fully automated techniques seen in Im2Calories and goFOOD™, which utilize advanced models like DeepLab and Mask R-CNN for precise segmentation. Meanwhile, advanced segmentation models such as Mask R-CNN and DeepLab, while achieving high accuracy in food recognition tasks, are computationally intensive, making them less suitable for mobile or real-time applications where efficiency is paramount. These models involve complex architectures with multiple layers and extensive parameter sets, resulting in significant processing time and memory requirements. Such computational demands can hinder their deployment on resource-constrained devices like smartphones or in scenarios requiring immediate responses. Addressing these limitations often necessitates the exploration of lightweight alternatives, such as MobileNet or YOLO-based frameworks, or applying optimization techniques like model pruning and quantization to improve the feasibility of using these advanced models in practical, real-time settings.

Applications like FoodLog and Snap-n-Eat adopt simpler, yet effective, block-wise analysis and saliency-based sampling for segmenting food items. Some, such as GoCarb and FoodLog, are optimized for the specific characteristics of food images, enhancing accuracy, while others like YOLOv2 in Food Tracker and the RPN in DeepFood use more general object detection frameworks. Interactive methods in goFOOD™ offer a balance between automation and user input, whereas fully automated approaches like NU-InNet and mobile food record (mFR) prioritize efficiency, especially in mobile contexts. Fine-grained segmentation in Im2Calories and DeepFood focuses on individual food items, while coarser methods like those in Snap-n-Eat are faster and suitable for broader region identification. Its mentionable Building on the foundation of the GoCARB system, the team introduced goFOOD™ (29). For semi-automatic segmentation, they continued utilizing region growing and merging algorithms. In addition, they developed a fully automated food segmentation method using Mask R-CNN (30). The recognition module was upgraded with an enhanced Inception V3 model, enabling more effective hierarchical food recognition. While GoCARB was designed primarily for carbohydrate calculation, goFOOD™ expands its functionality to estimate the calories and nutritional content of entire meals.

Recent advancements in image segmentation have introduced powerful methods like the Segment Anything Model (SAM) (31), which has gained significant attention for its versatility and accuracy. SAM, developed by Meta AI, is designed to handle a wide range of segmentation tasks with minimal fine-tuning, making it particularly useful for applications requiring high adaptability to diverse data types. Unlike traditional segmentation models that often require extensive training on specific datasets, SAM leverages prompt engineering to perform zero-shot segmentation across various domains, including medical imaging, object detection, and food image analysis. Its ability to generalize well across different tasks has set a new benchmark in segmentation accuracy and efficiency, outperforming earlier models in terms of both speed and precision.

By employing these segmentation techniques, food recognition applications can enhance their ability to provide accurate dietary assessments, offering users more reliable insights into their food intake. As the field continues to evolve, it is expected that further advancements in segmentation algorithms, particularly those powered by deep learning, will continue to improve the precision and usability of dietary assessment tools.

### 3.3 Image classification

Food image classification is a critical step in many food assessment applications, where the goal is to accurately identify and categorize food items from images. This process typically involves two main components: feature extraction and classification. Feature extraction involves identifying and quantifying the relevant attributes of an image, such as color, texture, and shape, which can then be used to distinguish different types of food. Classification refers to the process of assigning a label to the image based on these extracted features, determining the specific food item or category.

Traditional machine learning approaches to food image classification rely on manually engineered features and classical classifiers. In these methods, the feature extraction process involves using techniques such as edge detection, color histograms, and texture analysis to represent the image in a feature space. Once the features are extracted, classifiers like Support Vector Machines (SVM), k-Nearest Neighbors (k-NN), and Random Forests are employed to categorize the food items. These approaches require careful selection and design of features, which can be a time-consuming process, and often struggle with the variability and complexity of food images. The performance of traditional methods is also heavily dependent on the quality and relevance of the extracted features.

In contrast, deep learning approaches have revolutionized food image classification by automating the feature extraction process using convolutional neural networks (CNNs). CNNs can learn hierarchical features directly from the raw pixel data, capturing intricate patterns and relationships within the image that are often difficult to detect with traditional methods. This ability to learn from data has led to significant improvements in classification accuracy, particularly for complex and diverse food items. Deep learning models, such as those based on CNN architectures like AlexNet, ResNet, and Inception, are capable of handling large-scale datasets and can generalize well to new, unseen food items. These models have become the standard in food image classification, outperforming traditional approaches in both accuracy and scalability.

Table 4 highlights how food assessment applications employ diverse classification methods, from traditional machine learning to advanced deep learning, each tailored to specific tasks. PlateMate utilizes a manual, user-driven approach where food items are described and matched to a database, relying on crowdsourced voting to refine classification accuracy. This method, while interactive, is heavily dependent on user input, which may limit scalability and consistency.

**Table 4:** Classification strategies employed in food image processing applications.

App	Classification Method	Aproach
PlateMate (39)	Identify: Describing Items Matching Foods	Users describe each segmented food item in their own words, which are then matched to a database of foods. Then, they match the described items to entries in a commercial nutrition database. The classification is refined by additional voting and agreement detection among multiple users.
FoodLog (37)	Global Image Features AdaBoost for Food Category Classification	In addition to block-wise analysis, the entire image is analyzed using global features such as color, Bag of Features (BoF) from local features (like SIFT), and circle features (to detect round objects like plates). The image’s global feature vector is fed into an AdaBoost classifier that determines the class (or “Serving Value”—SV) of each food category present in the image.
Snap-n-Eat (36)	Feature Extraction Fisher Vector Encoding Support Vector Machine (SVM) Classifier	Low-level features such as (HOG) and (SIFT) are extracted from the segmented regions. These features capture the texture and shape information of the food items. The extracted features are encoded using the Fisher Vector method, which augments the traditional Bag of Visual Words (BoVW) by encoding higher-order statistics. This improves representation and classification accuracy. A linear SVM is then used to classify the segmented regions into different food categories. The system is trained on a dataset specifically collected for this purpose, consisting of 2,000 images across 15 food categories.
Menu-Match (42)	Bag of Visual Words Approach (Color, Histogram of Oriented Gradients (HOG), (SIFT), Local Binary Patterns (LBP), MR8 Filter Bank) Support Vector Machine (SVM)	These features are encoded using Locality-Constrained Linear Encoding (LLC) and pooled using a rotation-invariant pooling scheme across multiple scales to achieve robust feature representation. The pooled features are then used for classification through (SVM), which assigns scores to different food items based on the trained model.
Im2Calories (7)	Multi-label classifier based on a CNN (GoogLeNet). The model is pre-trained on the ImageNet dataset and then fine-tuned on various food datasets such as Food101 and a custom Restaurant dataset.	The classification framework is tailored to handle multiple food items on a plate, allowing for the prediction of the presence of several food items in an image. This multi-label classification system is crucial for identifying different components of a meal, which are then used for further processing, such as segmentation and volume estimation.
FoodCam (43)	Bag-of-Features (BoF) and Color Histograms with *χ*^2^ Kernel Feature Maps using Linear SVM. HOG Patch Descriptor and Color Patch Descriptor with Fisher Vector Representation.	This method combines the standard bag-of-features (using dense-sampled SURF descriptors) and a 64-bin RGB color histogram divided into 3 × 3 blocks. The features are then classified using a linear Support Vector Machine (SVM) with a fast χ2 kernel based on kernel feature maps. The second method uses Histogram of Oriented Gradients (HOG) patches and color patches, which are then encoded using Fisher Vectors. This method provides higher recognition accuracy with lower computational complexity, making it more suitable for mobile devices.
GoCarb (44, 45)	Feature Extraction: Classification using SVM: Performance: The classification method achieved an accuracy of 87–90% across different datasets and food classes.	Color Features: Histogram of the 1,024 most dominant colors, generated using hierarchical k-means clustering. Texture Features: Histogram of the 256 values of the local binary pattern (LBP). The combined color and texture feature vector (1,280 dimensions) is fed into a nonlinear support vector machine (SVM) with a radial basis function (RBF) kernel to assign the segment to one of nine predefined food classes.
NU-InNet (46)	NU-InNet Architecture: The paper introduces two versions of a CNN-based architecture called NU-InNet (Naresuan University Inception Network), designed for Thai food image recognition on smartphones.	NU-InNet 1.0: This architecture modifies the inception module from GoogLeNet by changing the 3×3 max-pooling layer and 1×1 convolutional layer to 1×1 and 7×7 convolutional layers, respectively. It includes 16 layers in total and is optimized for reducing processing time and model size while maintaining high accuracy. NU-InNet 1.1: This version further modifies NU-InNet 1.0 by replacing 5×5 convolutional layers with two 3×3 convolutional layers and 7×7 convolutional layers with three 3×3 convolutional layers. It includes 21 layers and aims to reduce processing time even further, while still offering high recognition accuracy.
DietLens (38)	ResNet-50 Model	The system employs the ResNet-50 deep learning model for food recognition. ResNet-50 consists of 50 convolutional layers and is fine-tuned on a dataset specifically constructed for Singapore hawker food. The system classifies food items into one of 249 categories, covering a diverse range of foods including Chinese, Western, Indian, and Malay dishes.
Food Tracker (47)	Deep Convolutional Neural Network (DCNN): The paper utilizes a DCNN model built upon MobileNet and adapted with the YOLOv2 detection framework.	The DCNN architecture is composed of 30 layers and 3.5 million parameters. It uses depth-wise separable convolution blocks to reduce computation costs and parameters, making it suitable for mobile applications. The model is trained to classify multiple food items simultaneously, achieving a mean average precision (mAP) of 76.36% on the UECFood100 dataset and 75.05% on the UECFood256 dataset.
MyDietCam (48)	Transfer Learning: ResNet-50, DenseNet201 and Inception ResNet-V2 Adaptive Reduced Class Incremental Kernel Extreme Learning Machine (ARCIKELM)	Deep learning models pre-trained on ImageNet are used for feature extraction. The 3 methods used to determine the best architecture for food feature extraction. This novel classifier is designed to handle class incremental and data incremental learning. It dynamically adjusts its architecture to reduce catastrophic forgetting and adapts to domain changes by adding new neurons as needed. It uses a kernel matrix for enhanced stability and generalization, unlike traditional ELM methods.
goFOOD™ (29)	Hierarchical Classification, (CNNs), specifically an Inception V3 model, to recognize food items at different levels of granularity, from broad categories to specific dishes	goFOOD™ supports 319 pre-installed fine-grained food categories organized into a three-level hierarchical architecture. The model predicts food categories at three levels, and the final classification is based on a weighted inference of these predictions.
DeepFood (49)	Deep Convolutional Neural Network (CNN): The classification of food items is conducted using a CNN, specifically the VGG-16 model.	The extracted feature maps from the RoIs are classified into different food categories. The classification process also involves a bounding box regression module to enhance the accuracy of the detected food regions in the image. The VGG-16 model uses a 4,096-dimensional feature vector to classify food items into categories, and the softmax layer contains 100 units for 100 food categories.
mobile food record (mFR) (50)	Hierarchical Food Classification Continual Learning	A two-step approach involving Convolutional Neural Networks (CNNs) is used to localize food items and then classify them hierarchically to reduce prediction errors, especially for visually similar foods. This approach is designed to handle online learning scenarios with limited data and real-time constraints, ensuring the system adapts over time.

In contrast, FoodLog and Snap-n-Eat employ more automated methods, using global image features and support vector machines (SVM) for classification. FoodLog combines block-wise analysis with global features like color histograms and Bag of Features (BoF), whereas Snap-n-Eat focuses on texture and shape information using HOG and SIFT descriptors, further enhanced by Fisher Vector encoding. These methods are more efficient but may struggle with complex food images where handcrafted features are insufficient to capture the necessary detail.

Deep learning approaches have significantly advanced the field of food image classification. For example, Im2Calories uses a CNN-based multi-label classifier, fine-tuned on large food datasets, to handle multiple food items in a single image. This method exemplifies the power of deep learning in capturing intricate patterns within food images, allowing for more accurate and scalable classification.

NU-InNet and Food Tracker further illustrate the effectiveness of deep learning, with architectures specifically optimized for mobile devices. NU-InNet, modifies the inception modules from GoogLeNet to balance processing time with accuracy, while Food Tracker uses a deep convolutional neural network (DCNN) based on MobileNet and YOLOv2, achieving impressive performance with minimal computational cost.

DietLens and MyDietCam leverage transfer learning, using pre-trained models like ResNet-50 and DenseNet201 to extract features, which are then classified using either traditional SVMs or innovative methods like ARCIKELM for adaptive learning. These approaches demonstrate how deep learning models, pre-trained on extensive datasets like ImageNet, can be adapted to specific food classification tasks with high accuracy.

Finally, applications like goFOOD™ and DeepFood highlight the utility of hierarchical classification and CNNs in handling fine-grained food categories. goFOOD™, employs a hierarchical classification scheme using an Inception V3 model to recognize food items at different levels of granularity, from broad categories to specific dishes. DeepFood utilizes the VGG-16 model, which combines region-based feature extraction with bounding box regression, ensuring precise classification even in complex food images.

One of the most recent classification approaches for food recognition involves the use of Vision Transformers (ViTs) (32) and Self-Supervised Learning (SSL) techniques (33). Vision Transformers, originally developed for natural language processing tasks (32), have been adapted for image classification and are gaining popularity due to their ability to capture long-range dependencies and global image context more effectively than traditional convolutional neural networks (CNNs). Self-Supervised Learning, on the other hand, leverages large amounts of unlabeled data to pre-train models, which are then fine-tuned on specific food datasets. This approach reduces the reliance on labeled data, which is often scarce in food recognition tasks, and improves the generalization capabilities of the model across different food categories.

Overall, the trend in food image classification is moving towards deep learning-based methods, which offer superior accuracy, scalability, and the ability to handle complex and diverse food images with minimal manual intervention. These methods have set a new benchmark in the field, outperforming traditional machine learning approaches, especially in terms of efficiency and adaptability to new data.

### 3.4 Volume estimation

Image volume estimation is a critical aspect of food recognition systems, particularly in dietary assessment applications. Accurate volume estimation allows these systems to determine the portion sizes of food items, which is essential for calculating nutritional intake, including calories, macronutrients, and micronutrients. The challenge in estimating food volume from images lies in the inherent variability in food presentation, such as different shapes, sizes, and textures, as well as varying camera angles and lighting conditions.

Several methods have been developed to estimate food volume from images, ranging from traditional geometric approaches to advanced machine learning techniques. Geometric methods typically involve using reference objects (like a standard-sized plate or utensil) to scale the food item in the image, enabling volume calculations based on known shapes (e.g., spheres, cylinders). On the other hand, machine learning approaches often leverage deep learning models trained on large datasets of food images to estimate volume directly from pixel data.

Table 5 highlights the diversity of methods used for volume estimation in food recognition applications.

**Table 5:** Volume estimation strategies employed in 14 food image processing applications.

App	Volume Estimation Method	Approach
PlateMate (39)	Aggregating Measurements	Turkers estimate the portion size of each identified food item. They choose appropriate units (e.g., slices, cups) and provide a numerical estimate. The system uses these estimates to calculate nutritional content, with multiple Turkers providing input to reduce errors through averaging and outlier removal.
FoodLog (37)	Food Balance Estimation Bayesian Framework for Personalization	The system estimates the food balance by evaluating the proportion of each food category in the image. This is done by summing the SVs from the classified blocks and comparing them against a reference food pyramid model. The system allows for user feedback to incrementally update and personalize the classification model using a Bayesian framework, which refines the estimation by incorporating user-specific dietary tendencies.
Snap-n-Eat (36)	Pixel Counting: Caloric and Nutritional Estimation:	The system estimates the portion size of each food item by counting the number of pixels in its corresponding segment. This simple approach provides a reasonably accurate estimate of the portion size. Once the portion size is determined, the system calculates the caloric and nutritional content based on predefined caloric and nutrition densities for each food category.
Menu-Match (42)	The paper proposes a method that circumvents the traditional need for direct volume estimation by leveraging the consistent serving sizes of restaurant meals.	The restaurant-specific database includes nutritional information for predefined items, volume estimation is inherently addressed by identifying the food item and referring to its known nutritional content in the database. This reduces the problem to identifying the correct food item and applying pre-recorded values, avoiding the complexities of estimating volume from an image.
Im2Calories (7)	Predicting the depth of each pixel using a CNN architecture similar to the one described by Eigen and Fergus in 2014 (78).	CNN is trained on the NYU v2 RGBD dataset (28) of indoor scenes and fine-tuned on a new 3D food dataset called GFood3D. Once the depth map is predicted, it is converted into a voxel representation by projecting each pixel into 3D space, considering the table surface detected using RANSAC. The final step involves calculating the volume of each food item by estimating the average height of points in each cell of the voxel grid.
FoodCam (43)	Relies on user input	After recognizing food items within the bounding boxes, the system allows users to estimate the rough volume of the food item using a slider on the screen. This interaction is part of the user-assisted approach to volume estimation, which complements the food recognition process.
GoCarb (44, 45)	Pose Estimation: Scale Extraction: D Reconstruction: Volume Calculation: Accuracy: The volume estimation method achieved a mean absolute percentage error of 9.4%.	As input A pair of images from different viewing angles, both including a reference card. Speeded up robust features (SURF) are detected and matched between the two images. RANSAC model fitting is used to extract a candidate pose, optimized through iterative minimization. The reference card’s known dimensions are used to extract the scale of the scene. Polar rectification transforms the image pairs so that corresponding points lie on the same row, facilitating efficient point matching. The unprojection of correspondence yields a scaled 3D point cloud that defines the food surface. The food item’s volume is calculated by partitioning the surface according to its projection on the segmentation map and integrating the space between each subsurface and the plate surface.
NU-InNet (46)	The paper does not explicitly discuss methods for volume estimation. The focus is primarily on the accuracy and efficiency of food recognition using CNNs on mobile devices.	Volume estimation would require additional steps not covered in this paper
DietLens (38)	Photo-Based Volume Estimation	The volume or portion size estimation in DietLens is handled through a reference-based approach, where users select the closest matching portion size from a set of reference images. This method is integrated with nutritional databases to provide the corresponding nutritional information based on the selected portion size. This photo-based estimation method eliminates the need for traditional volume estimation techniques and instead relies on visual comparison with pre-determined portion sizes.
Food Tracker (47)	Not specified	The application assumes that each detected food item represents one serving and retrieves nutrition information for that serving from the Nutritionix database. The paper indicates that future work will focus on mask generation for more accurate volume estimation, which suggests that this aspect is still under development.
MyDietCam (48)	Not specified	
goFOOD™ (29)	3D reconstruction-based approach, Sensor-Assisted	When using different-view images from a single-camera smartphone, a reference object of known size is required. The method builds on previous work from the GoCARB system but improves accuracy by integrating gravity data from the smartphone’s Inertial Measurement Unit (IMU). This data helps determine the orientation of the table and simplifies camera pose estimation, leading to more stable and accurate volume reconstruction.
DeepFood (49)	The paper focuses on calculating the nutritional contents, including calories, fats, carbohydrates, and proteins, from each meal image. However, specific volume estimation methods for determining the physical volume of food items are not discussed in detail. The system estimates nutrition based on standard portion sizes, assuming a default weight (e.g., 400 grams) for each detected food item.	Nutritional analysis is then performed by mapping the detected food to a reference table of nutrition facts using data from the USDA National Nutrient Database (NNDB). So, volume estimation is not explicitly addressed.
mobile food record (mFR) (50)	Geometric Models Deep Learning	Single-view image analysis is employed to recover 3D parameters of food objects in the scene, aiding in portion size estimation. Deep learning techniques are used to map food images directly to food energy estimates, enhancing the accuracy of portion size estimation.

The volume estimation methods across these food assessment applications vary significantly in complexity and accuracy. PlateMate and FoodLog utilize crowd-sourced input and Bayesian personalization, respectively, which can improve accuracy but are dependent on user input and initial classification quality. Snap-n-Eat and DietLens use simpler techniques like pixel counting and reference images, respectively, making them user-friendly but potentially less precise. Menu-Match avoids direct volume estimation by relying on predefined data, which simplifies the process but limits its applicability to custom meals. Im2Calories and GoCarb employ advanced 3D reconstruction and pose estimation methods, providing high accuracy but requiring complex setups and multiple images. FoodCam relies on user input for volume estimation, which can be inconsistent. NU-InNet does not address volume estimation, focusing instead on food recognition accuracy. MyDietCam lacks specific details on its volume estimation method. goFOOD™ combines 3D reconstruction with gravity data for improved accuracy, though it requires additional hardware (sensor assisted volume estimation). DeepFood estimates nutritional content without specific volume estimation, assuming standard portion sizes. Finally, mobile food record (mFR) uses a combination of geometric models and deep learning for portion size estimation, offering a sophisticated approach but demanding significant computational resources. Overall, methods like Im2Calories and GoCarb are superior in terms of accuracy due to their advanced techniques, while applications like NU-InNet and MyDietCam fall short by not presenting specific volume estimation methods.

One of the recent advancements in volume estimation processes is FOODCAM 2022 (34), an imaging-based method specifically designed for food portion size estimation (FPSE). It employs a novel capturing device that delivers greater accuracy compared to traditional methods. The system integrates a stereo camera, PIR sensor, and infrared projector, enabling precise meal portion size estimation. FOODCAM was primarily designed for monitoring food intake and cooking activities in kitchen and dining environments.

Since the focus of FOODCAM 2022 is exclusively on portion size estimation, it was excluded from our discussion on food recognition applications. It’s important to note that FOODCAM 2022 is distinct from the FoodCam calorie-counting application developed in 2015, which was included in the tables. While both share the name “FoodCam,” the FOODCAM 2022 device is dedicated to volume estimation, whereas the FoodCam application developed in 2015 focuses on calorie tracking.

Accurate volume estimation in most of these apps relies on user input for near-perfect estimation, which can be prone to human measurement inaccuracies. A parallel challenge arises in automated food volume estimation, where overfitting becomes a critical issue, especially when models are trained on narrow or non-representative datasets that fail to capture the full diversity of real-world conditions. For instance, many existing volume estimation methods inadvertently memorize dataset-specific artifacts (e.g., background noise, lighting conditions, or food presentation styles) rather than learning generalizable features. This over-reliance on training data idiosyncrasies results in poor performance when deployed in variable, uncontrolled environments, compromising their accuracy in practical applications.

## 4 Ethical and privacy concerns

The ethical implications of AI-driven dietary monitoring tools are significant, particularly in the areas of data privacy, potential biases, and transparency. Sensitive dietary and health data collected by these applications must comply with stringent data protection frameworks, such as the General Data Protection Regulation (GDPR), which emphasize anonymization, secure storage, and encryption to mitigate the risks of misuse. Furthermore, biases stemming from imbalanced datasets, which may inadequately represent diverse cultural and demographic contexts, pose challenges to model accuracy and fairness. To address this, the diversification of datasets and the implementation of regular fairness audits are essential for achieving equitable outcomes. Transparency and accountability also play a vital role; explainable AI (XAI) techniques can elucidate model decision-making processes, fostering user trust and confidence in technology. As highlighted in recent literature (35), adopting comprehensive ethical guidelines and promoting collaboration among stakeholders are critical steps in addressing risks. These efforts, combined with robust audit mechanisms and user education programs, ensure the responsible development and deployment of AI-driven dietary monitoring tools.

## 5 App store availability, user ratings, and performance metrics

To gauge real-world user adoption and satisfaction, we conducted a search for these applications in major consumer app stores (Google Play and Apple’s App Store). However, we found that most of the listed calorie-counting solutions are academic prototypes or research-oriented tools rather than commercial products. Consequently, they are not publicly available in mainstream app stores, and official user ratings are unavailable. Some studies [e.g., Snap-n-Eat (36), FoodLog (37)] do discuss small-scale pilot tests or user acceptance evaluations within controlled research settings, but these do not constitute widespread consumer feedback akin to star ratings or download counts. Where a limited pilot or spin-off was mentioned (e.g., DietLens (38)), we could not locate any corresponding listing under the same app name. These findings highlight the research-focused nature of most solutions, emphasizing the need for future work on broader deployment, real-world user engagement, and the potential transition to public app store availability.

Our primary emphasis, however, is on the computer science methodologies underpinning these applications. By examining their underlying algorithms, we can better determine how effectively each solution tackles existing challenges, such as accurate portion estimation and real-time processing. Focusing on these methodological aspects allows us to identify limitations and highlight advantages relevant to the future design and deployment of calorie-counting applications.

Table 6 summarizes the reported performance metrics for each calorie-counting or food recognition application, as documented in their respective publications. Whenever possible, we include classification accuracy, mean error rates, or other relevant statistics. Despite differences in datasets and validation protocols, the reported accuracy and error rates provide insight into how well each application addresses calorie estimation or food recognition. For instance, PlateMate overestimates caloric content by +7.4%, which is close to the +5.5% error reported for a trained dietitian in the same study (39). Classification-based approaches generally report accuracy metrics in the 70–90% range, but they are measured on diverse datasets (e.g., Food-101, UEC-Food256), making direct comparisons challenging. Some solutions, such as Menu-Match and DeepFood, achieve relatively high Top5 accuracies of over 90% on specific datasets. Others, like FoodCam, show a lower Top1 accuracy (around 50%), yet still improve markedly with Top5 predictions (74.4%). Meanwhile, MyDietCam notes strong results of over 80% across multiple datasets, but 100% accuracy in certain controlled conditions (PFID dataset). Overall, although performance varies by dataset and study design, these figures highlight the progress in automated food recognition and the ongoing need to refine algorithms for real-world calorie counting applications.

**Table 6:** Reported performance metrics in 14 food image processing applications.

App	Performance metric
PlateMate (39)	Average caloric estimation error: +7.4% (overestimation)
FoodLog (37)	Food-image detection accuracy: 89–92%
Snap-n-Eat (36)	Classification accuracy: 85%
Menu-Match (42)	Classification accuracy: ChineseFoodNet = 77.4%(Top1) and 96.2% (Top5)
Im2Calories (7)	Classification accuracy: Food-101 dataset: 79% (Top1)
	Segmentation accuracy: Food-101 dataset 71–76%
FoodCam (10, 43)	Classification accuracy: UEC-Food256 dataset: 50.1%(Top1) and 74.4% (Top5)
GoCarb (44, 45)	Classification accuracy: 87–90% (various datasets and food classes).
	Volume estimation: (mAP) of 9.4%.
NU-InNet (46)	Classification accuracy: 10 THFOOD-50 = 69.8%
DietLens (38)	Average recognition accuracy: 75.2% (Top1) and 93.1% (Top5)
Food Tracker (47)	Classification accuracy: (mAP) of 76.36% on the UECFood100 dataset and 75.05% on the UECFood256 dataset.
MyDietCam (48)	Classification accuracy:
	Food-101 = 87.3%
	UECFOOD-100 = 88.7%
	UECFOOD-256 = 76.51%
	PFID = 100%
	Pakistani Food = 74.8%
goFOOD™ (29)	Classification accuracy: 65.8%(Top1) and 82.4%(Top3)
DeepFood (49)	Classification accuracy: 71.7% (Top1) and 93.1% (Top5) # at 500 iterations
mobile food record (mFR) (50)	Mean error rate: 11.22%.

## 6 Key findings and remaining challenges

This review sheds light on key aspects of image-based food monitoring systems, focusing on segmentation, classification, and volume estimation techniques used in calorie-counting applications. It highlights recent advancements in deep learning and image processing that have significantly improved the accuracy of food recognition and dietary intake estimation. However, challenges such as standardization, validation across diverse food types, and privacy concerns remain significant hurdles.

As technology continues to advance, the integration of machine learning and computer vision techniques is expected to further enhance the accuracy and reliability of food volume estimation in dietary assessment applications. These advancements will contribute to the development of more sophisticated tools for personalized nutrition and health management, providing users with detailed insights into their dietary habits and improving overall outcomes. Despite this progress, the review identifies that many applications still rely on user input and suffer from inconsistencies in image quality, highlighting the need for continued innovation.

To address these challenges, this review presents the following recommendations aimed at enhancing digital remote healthcare for patients with diabetes and weight-related chronic diseases:

- Develop robust, standardized algorithms capable of handling a wide range of food types and presentation styles.
- Eenhancing volume estimation techniques to minimize reliance on user input and reduce the need for specialized hardware will further enhance the accessibility and accuracy of next-generation calorie-counting systems.
- Prioritize secure, privacy-preserving methods for managing sensitive dietary and health-related data.
- Collaborate with healthcare professionals to ensure tools provide effective support for patient education, health behavior monitoring, and personalized dietary recommendations.
- Collaborate with patients living with T2D to ensure feasibility, acceptability, ease of navigation, and appropriateness of tools and supportive material, including education and support.

### 6.1 Future opportunities in emerging technologies

As AI-based architecture continues to evolve, new opportunities arise to enhance the accuracy, scalability, and usability of calorie-counting applications while maintaining user-friendliness. For instance, 3D sensing technologies, such as LiDAR sensors integrated into Apple smartphones, can be leveraged to improve the precision of volume estimation modules. These sensors provide high-resolution depth information, enabling more reliable food volume assessments. Additionally, advanced computational approaches for 3D reconstruction, for instance, monocular depth estimation (40), offer promising alternatives by generating detailed three-dimensional representations from single or multiple images. Incorporating such techniques can enhance model robustness across diverse food types and real-world conditions, further improving the reliability of dietary assessment tools.

To address the generalizability issues affecting most current calorie-counting applications, federated learning (41) can be explored as a promising approach. By enabling image classification and volume estimation models to be trained collaboratively across multiple user devices while keeping data local, federated learning enhances the robustness of the global model. This approach not only improves generalization across diverse food types and real-world conditions but also allows for personalization without compromising user privacy. Additionally, it reduces the risk of data centralization vulnerabilities while leveraging distributed computing to adapt models to individual dietary habits and variations in food presentation.

The challenges and limitations identified in this research, along with the proposed future directions, pave the way for the development of a new generation of calorie-counting applications. These next-generation applications can leverage cutting-edge techniques to address key issues such as real-time performance, accuracy across diverse dietary scenarios, and user-specific personalization. By overcoming these limitations, future calorie-counting solutions will become more reliable, user-friendly, and seamlessly integrated into clinical settings, ultimately supporting dietary management and chronic disease prevention.

## 7 Conclusion

In conclusion, this review underscores the critical role of advanced computer science techniques in enhancing the accuracy and effectiveness of calorie-counting applications. By evaluating the methodologies employed in existing systems, we have identified both their strengths and areas in need of improvement. This work represents a significant step forward in the field, contributing to the ongoing evolution of digital health tools aimed at better managing dietary intake. The insights gained from this review will guide the development of a new, state-of-the-art calorie-counting app designed to address the existing challenges and provide more reliable, personalized dietary assessments to support patients living with T2D and their clinicians in the international fight against T2D.
